# Reduced Responsiveness to Long-Term Monocular Deprivation of Parvalbumin Neurons Assessed by c-Fos Staining in Rat Visual Cortex

**DOI:** 10.1371/journal.pone.0004342

**Published:** 2009-02-04

**Authors:** Marco Mainardi, Silvia Landi, Nicoletta Berardi, Lamberto Maffei, Tommaso Pizzorusso

**Affiliations:** 1 Laboratory of Neurobiology, Scuola Normale Superiore, Pisa, Italy; 2 Department of Psychology, University of Florence, Florence, Italy; 3 Institute of Neuroscience, CNR, Pisa, Italy; James Cook University, Australia

## Abstract

**Background:**

It is generally assumed that visual cortical cells homogeneously shift their ocular dominance (OD) in response to monocular deprivation (MD), however little experimental evidence directly supports this notion. By using immunohistochemistry for the activity-dependent markers c-Fos and Arc, coupled with staining for markers of inhibitory cortical sub-populations, we studied whether long-term MD initiated at P21 differentially affects visual response of inhibitory neurons in rat binocular primary visual cortex.

**Methodology/Principal Findings:**

The inhibitory markers GAD67, parvalbumin (PV), calbindin (CB) and calretinin (CR) were used. Visually activated Arc did not colocalize with PV and was discarded from further studies. MD decreased visually induced c-Fos activation in GAD67 and CR positive neurons. The CB population responded to MD with a decrease of CB expression, while PV cells did not show any effect of MD on c-Fos expression. The persistence of c-Fos expression induced by deprived eye stimulation in PV cells is not likely to be due to a particularly low threshold for activity-dependent c-Fos induction. Indeed, c-Fos induction by increasing concentrations of the GABAA antagonist picrotoxin in visual cortical slices was similar between PV cells and the other cortical neurons.

**Conclusion:**

These data indicate that PV cells are particularly refractory to MD, suggesting that different cortical subpopulation may show different response to MD.

## Introduction

It is well known that MD shifts the OD of neurons in the binocular portion of the primary visual cortex in favour of the nondeprived eye during a sensitive period [1]. However, it is still poorly understood whether all cortical cells shift similarly in response to MD. Recent data have shown that some cortical cells shift their response in favour of the deprived eye, with the direction of the shift critically depending on the amount of open-eye input and the net visual drive experienced during MD [2]. Studies in cortical slices also show cell-specific effects of MD. For instance, visual deprivation can induce a cell-specific potentiation in the circuit between fast-spiking inhibitory cells and pyramidal neurons [3].

Inhibitory neurons have been involved in the mechanisms controlling visual cortical plasticity by several studies [4]. It has been suggested that the effect of MD depends on the balance between excitation and inhibition, with the inhibitory circuitry acting as a “plasticity gate” over the excitatory components [5]. Moreover, interfering with inhibitory function alters the response to MD: mice with a genetic deletion of the GAD65 isoform of the GABA-synthesizing enzyme have no OD shift after MD, and this deficit can be restored by enhancement of inhibition with benzodiazepines [6]. Therefore, it is possible that MD alters the excitation/inhibition balance by acting differentially on inhibitory neuronal populations. Indeed, a recent *in vivo* study of visual calcium responses showed a delayed effect of MD on inhibitory neurons labelled by GFP knocked in the *GAD67* gene [7]. However, no *in vivo* study has investigated whether MD differentially affects the visual response of the various classes of GABAergic cells.

Many subpopulations of inhibitory interneurons can be identified in the cerebral cortex with a good overlap between neurochemical, anatomical and electrophysiological criteria [8]. In particular, inhibitory interneurons can be classified according to the diverse expression of Ca-binding proteins, identifying three mainly non-overlapping populations, containing respectively parvalbumin (PV), calbindin (CB), or calretinin (CR) [9], [10]. In the present work we analyze the effects of MD on the cells positive for the general marker of inhibitory neurons GAD67 or for the specific markers PV, CB and CR, using an anatomical approach based on the activity-dependent expression of Arc [11] and c-Fos [12]. While Arc showed little co-localization with inhibitory interneurons even after visual stimulation, c-Fos expression was clearly induced by visual stimulation in inhibitory cells. c-Fos induction is a good marker of suprathreshold neuronal activation [13], [14] that was previously used to assess MD effects [12]. Therefore, we adopted c-Fos staining coupled to GAD67, PV, CB and CR staining after visual stimulation of a long-term deprived eye to investigate MD effects on neuronal populations expressing these markers. We found that MD affected GAD67 and CR positive cells by reducing visual induction of c-Fos. CB positive neurons responded to MD by reducing CB expression. In contrary, MD did not affect visual activation of c-Fos in PV cells.

## Results

### c-Fos is a marker of the cortical effects of long-term MD on the inhibitory circuitry

**Figure 1:**
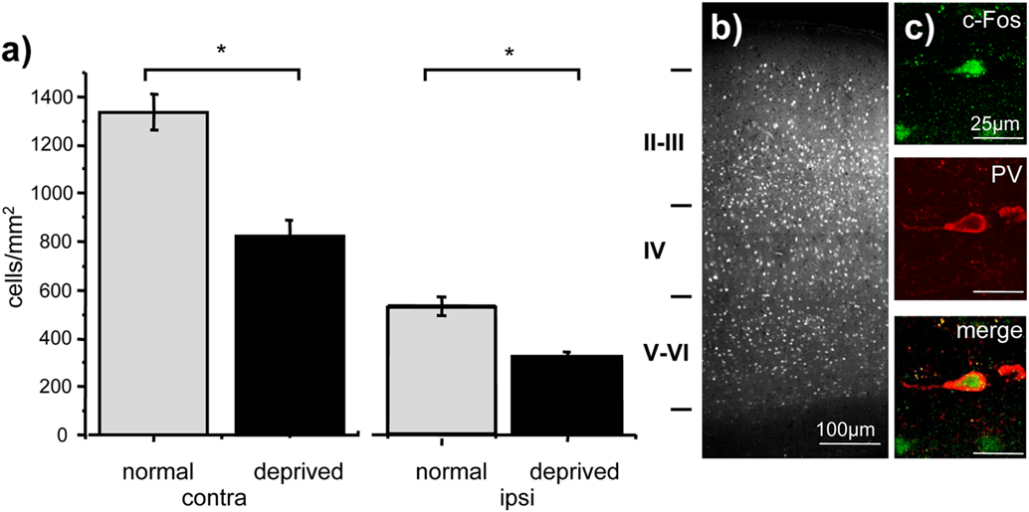
Visual activation of c-Fos. a) c-Fos activation by light is decreased in the binocular primary visual cortex of deprived animals both in the cortex contralateral (*t* test, P<0.001, N = 11) and ipsilateral (*t* test, P<0.001, N = 11) to the deprived eye; b) example of c-Fos staining after visual stimulation; c) high-magnification images showing the typical co-localization between c-Fos and PV.

To study the effects of long-term MD on the visual response of specific cortical interneurons, we used immunohistochemistry for the activity-dependent markers c-Fos and Arc. Arc was increased by monocular visual stimulation in the visual cortex contralateral to the stimulated eye (483.61±48.60 cells/mm^2^ in the contralateral cortex vs. 123.49±17.47 cells/mm^2^ in the ipsilateral cortex; paired *t* test, P = 0.008), but it showed very little co-localization with PV immunoreactivity (Fig. S1). For this reason Arc was not used in our further analyses. By contrast, c-Fos activation was light-dependent and co-localized with PV cells as well as with CB, CR and GAD67 labelling (Fig. 1). Thus, c-Fos can be used to assess the effects of MD on inhibitory circuits.

**Figure 2:**
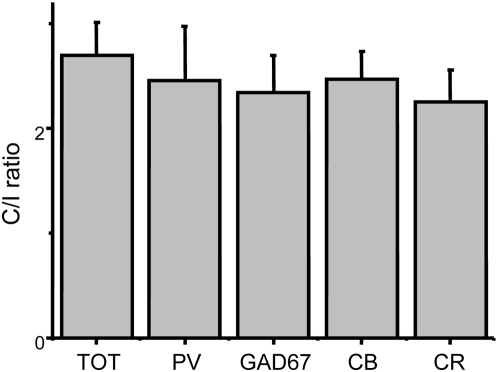
Contra/Ipsi ratio (C/I) of visual c-Fos induction in cortical neurons. The ratio between the number of c-Fos positive cells activated by contralateral and ipsilateral stimulation is reported. The TOT bar corresponds to the total cortical population. The other columns report the data for c-Fos positive cells double-stained with GAD67, PV, CB or CR. No significant difference is present (one way ANOVA, P = 0.952).

It is well known that the contralateral pathway is prevalent in driving the response of visual cortical cells in rodents [15], [16]. To study whether this contralateral bias is also present in visual c-Fos activation within the different subpopulations of inhibitory neurons, we analyzed the strength of the rat ipsilateral and the contralateral pathways in activating c-Fos in cells positive for GAD67, CB, CR and PV. Visual c-Fos induction cannot be used to assess ocular dominance at single cell level, however the measure of c-Fos activation by visual stimulation of the contralateral and the ipsilateral pathways is a measure of the overall binocularity. Therefore, we measured population binocularity by calculating the ratio (C/I ratio) between the number of c-Fos positive cortical cells detected after stimulation of the contralateral eye and the number of c-Fos positive cells detected after stimulation of the ipsilateral eye. The C/I ratio was initially calculated for the entire cortical population (fig. 2). The results showed that visual stimulation of the contralateral eye activated c-Fos in about twice the number of cells observed with ipsilateral eye stimulation. We then calculated the C/I ratio for the c-Fos labelled cells that were also positive for GAD67, CB, CR or PV. The C/I ratio within these inhibitory subpopulations was not different from the C/I ratio obtained for the entire cortical population (fig. 2), indicating that binocularity of these neuronal subpopulations is similar to the average binocularity of cortical cells.

### PV cells are distinctively refractory to MD

To evaluate the effects of long-term MD on c-Fos activation in inhibitory cells, we sutured the eyelids of one eye in P21 rats. After P150, we performed a monocular visual stimulation protocol and we assessed c-Fos activation. The results were compared with the effects of the same stimulation protocol in rats with previously normal visual experience. Monocular visual stimulation was achieved by reopening the deprived eye, while the fellow non-deprived eye was sutured, and immediately placing the animal in darkness for two days. The animal was then re-exposed to light for two hours.

As expected, MD decreased visual induction of c-Fos through the deprived eye (Fig. 1). In the cortex contralateral to the long-term deprived eye the density of c-Fos positive cells was lower (823.36±48.03 cells/mm^2^) than in the hemisphere contralateral to the stimulated eye of control animals (1335.67±75.62 cells/mm^2^; *t* test, P<0.001, N = 11). A similar result was observed in the hemisphere ipsilateral to the open eye, even if the absolute level of c-Fos expression was lower than in the contralateral hemisphere (MD 324.93±17.36 cells/mm^2^ versus control 548.99±39.24 cells/mm^2^; *t* test, P = 0.005, N = 11), in agreement with the relatively minor contribution of the ipsilateral pathway to the binocular visual cortex input.

**Figure 3:**
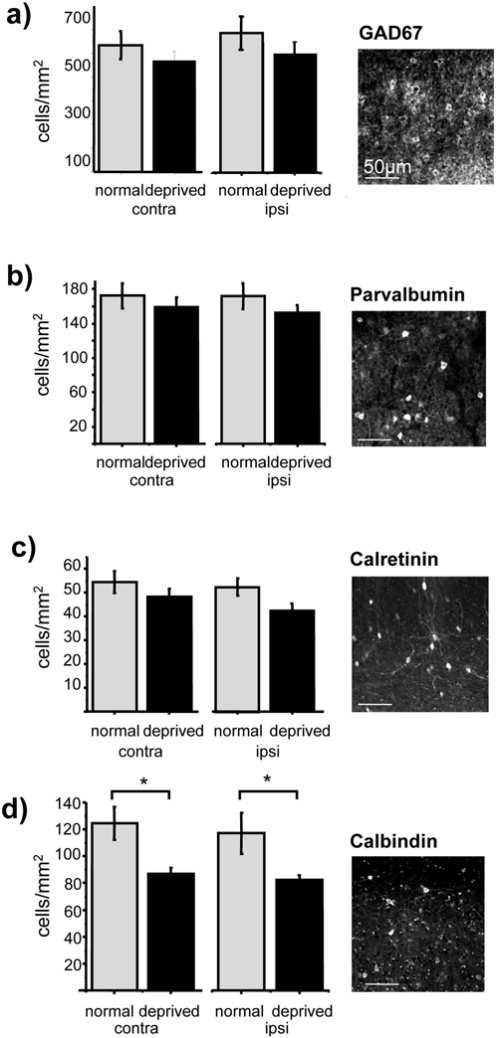
Effects of MD on inhibitory neuron markers. Long-term MD has no effect (*t* test, p>0.05) on the expression of GAD67 (a), PV (b) and CR (c), while it decreases the number of CB immunoreactive cells (d) in both hemispheres (*t* test, contra to the deprived eye P = 0.005, and ipsi P = 0.019).

Before assessing c-Fos activation in the inhibitory subpopulations identified by GAD67, PV, CB and CR labelling, we investigated whether long-term MD affected the number of cells expressing these markers (Fig. 3). There was not a statistically significant difference in the number of GAD67, CR and PV positive cells in long-term MD rats versus control, while the number of CB positive cells was reduced by MD in both hemispheres (contra, MD 86.90±4.62 cells/mm^2^ versus control 124.47±12.03 cells/mm^2^; *t* test, P = 0.005; ipsi, MD 82.34±5.39 cells/mm^2^ versus control 117.23±15.22 cells/mm^2^; *t* test, P = 0.019, N = 10).

Then, we counted cells double stained for c-Fos and the inhibitory neuron markers (GAD67, PV, CB and CR) in MD and control rats. Two different ratios were computed:

1. D/M = number of cells double stained for c-Fos and an inhibitory neuron marker/number of cells expressing the same inhibitory neuron marker;
2. D/F = number of cells double stained for c-Fos and an inhibitory neuron marker/number of c-Fos positive cells.

The D/M ratio expresses the strength of c-Fos induction within a cellular subpopulation. If MD is effective in reducing c-Fos induction in the subpopulation, but not in affecting the expression of the inhibitory neuron marker, this ratio should be reduced by MD. The D/F ratio indicates the contribution of a cell subpopulation to the overall c-Fos visual response of the cortex. If MD reduces c-Fos activation in this subpopulation similarly to the overall reduction observed in the cortical population, then the D/F ratio should not change.

**Figure 4:**
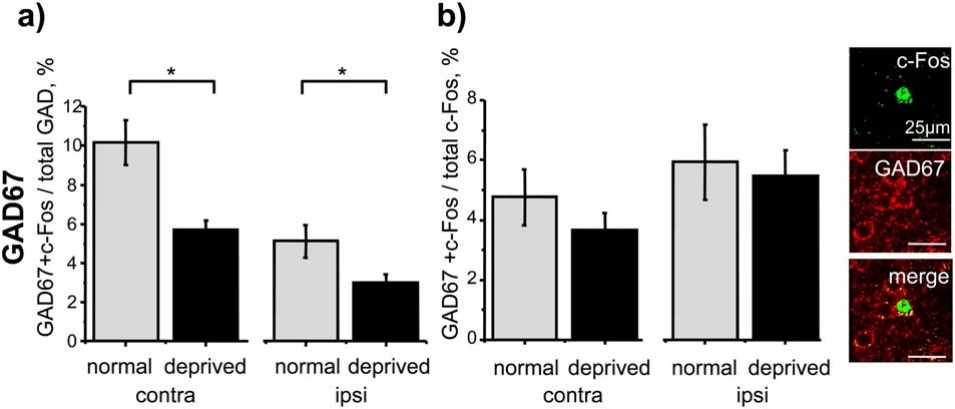
Effect of long-term MD on the whole population of cortical inhibitory interneurons. a) GAD67 cells show a reduction in the D/M ratio in both hemispheres of MD animals (contra, *t* test, P = 0.002; ipsi, *t* test, P = 0.037, N = 10); b) no difference is present in the D/F ratio of GAD67 cells between MD and control animals (contra, *t* test, P = 0.33; ipsi, *t* test, P = 0.76; N = 10).

We first evaluated the effect of long-term MD on cells expressing the general marker of inhibitory neurons GAD67. As it can be seen in Fig. 4a, the D/M ratio was reduced by MD in both hemispheres (contra, MD 5.70%±0.49 versus control 10.18%±1.14; *t* test, P = 0.002; ipsi, MD 3.03%±0.43 versus control 5.12±0.83; *t* test P = 0.037, N = 10). On the other hand, the D/F ratio was not significantly different in MD versus control rats (Fig. 4b; contra, *t* test, P = 0.33; ipsi, *t* test, P = 0.76), suggesting that the response of GAD67 positive neurons was reduced by MD.

**Figure 5:**
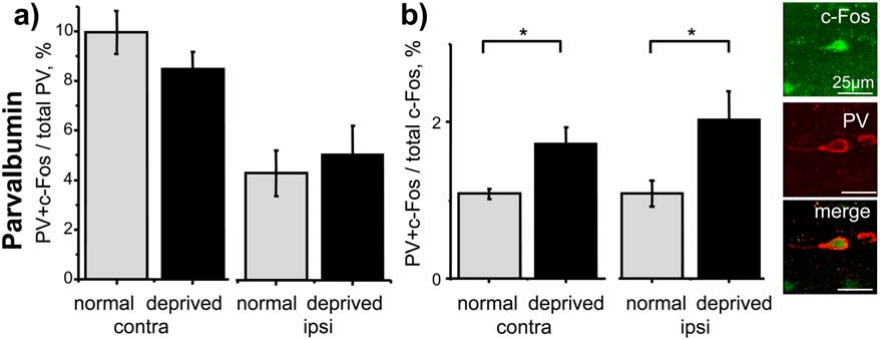
Effect of long-term MD on PV interneurons. a) PV interneurons did not show a significant decrease in the D/M ratio after long-term MD (contra, *t* test, P = 0.19; ipsi, *t* test, P = 0.65; N = 11), indeed b) the proportion of c-Fos positive cells which were also PVergic (D/F ratio) was increased in MD rats (contra, *t* test, P = 0.017; ipsi, *t* test, P = 0.041; N = 11).

We then analyzed the effects of MD on PV, CR and CB positive cortical inhibitory interneurons. Intriguingly, MD did not affect visual activation of c-Fos in PV neurons. The D/M ratio observed in MD animals was not different from that of control non-deprived animals, both in the hemisphere contralateral and ipsilateral to the deprived eye (Fig. 5a; contra, *t* test, P = 0.19; ipsi, *t* test, P = 0.65). Accordingly, the D/F ratio was significantly higher in deprived versus control animals in both hemispheres (Fig. 5b; contra, MD 1.72%±0.21 versus control 1.09%±0.06; *t* test, P = 0.017; ipsi, MD 2.03%±0.36 versus control 1.09%±0.17; *t* test, P = 0.041, N = 11), indicating that the long-term deprived eye retained its ability to activate cortical PV neurons.

**Figure 6:**
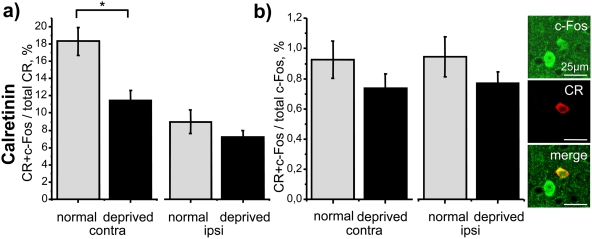
Effect of long-term MD on calretinin interneurons. CR interneurons show an analogous behaviour to the whole GABAergic population with a) a reduction in the D/M ratio (contra, *t* test, P = 0.003; N = 10) and b) lack of difference in the D/F ratio respect to control.

Similarly to GAD67 cells, in CR expressing neurons visual induction of c-Fos was strongly affected by MD. Indeed, MD induced a significant decrease of the D/M ratio in deprived versus control rats (Fig. 6a; MD 11.48%±1.16 versus control 18.31%±1.62; *t* test, P = 0.003, N = 10) in the hemisphere contralateral to the deprived eye. A similar trend, even if not statistically significant (*t* test, P = 0.24), was also observed in the ipsilateral cortex. In agreement with the responsiveness of CR cells to MD, the D/F ratio was unaltered by MD in both hemispheres (Fig. 6b; contra, *t* test, P = 0.23; ipsi, *t* test, P = 0.24).

The number of CB expressing neurons double stained for c-Fos was significantly reduced in MD animals with respect to normal rats (Fig. S2). However, the fact that the number of CB positive cells was also reduced by MD, and the impossibility to assess deprived eye responsiveness of the cells that have lost CB expression, did not allow to conclude that c-Fos expression in the total population of CB neurons is affected by MD. Indeed, it could be that neurons losing CB expression were still functionally preserved, retaining their capability to activate c-Fos in response to visual stimulation of the deprived eye. Thus, a consequence of MD on CB cells was the reduction of CB expression, but it is not known whether they also reduced their visual c-Fos activation.

In summary, MD decreases visually induced c-Fos activation in GAD67 and CR positive neurons. In the CB population MD decreased CB expression, while PV cells did not show any effect of MD on PV or c-Fos expression. Thus, PV cells are uniquely refractory to MD among the principal classes of cortical inhibitory neurons.

### PV cells do not have a specifically low threshold for pharmacological activation of c-Fos

**Figure 7:**
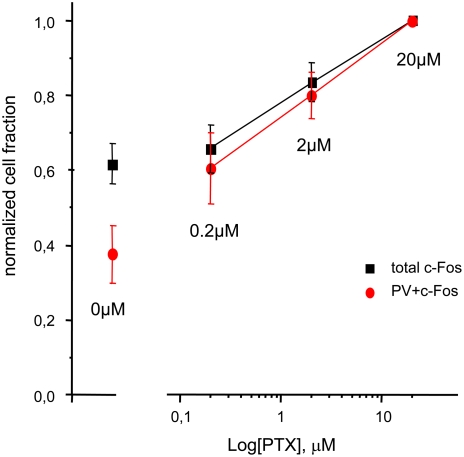
PV cells show c-Fos activation by PTX similar to the other cortical neurons. Dose-response curves after exposure to increasing concentrations (0, 0.2, 2, 20 µM) of PTX, showing comparable increases in c-Fos activation in the whole cortical cellular population (black) and in PVergic cells (red) (two-way ANOVA, effect of PTX concentration is significant with P = 0.003, while no significant effect of cell type and no interaction between cell type and PTX dose was present, P>0.05, N = 4). Data are normalized to the value obtained with stimulation with 20 µM PTX.

To check whether the permanency of the response of PV cells to stimulation of the deprived eye was due to a reduced threshold for activity-dependent induction of c-Fos in these cells, we perfused cortical slices with physiological solution containing increasing concentrations of the GABA_A_ antagonist picrotoxin. The total number of c-Fos positive cells and of double stained PV/c-Fos cells was counted. No difference was present between the dose-response curves of PV cells and that of all cortical neurons (Fig. 7). These data indicate that the lack of effect of MD on PV neurons is not due to a specifically low threshold for c-Fos activation in this cell population, but it is more likely due to a different effect of MD on PV cells and their inputs.

## Discussion

We have investigated the effects of MD on cortical inhibitory neurons expressing the general marker GAD67 or specific markers (PV, CB and CR) of the major GABAergic subpopulations. The effects of MD were assessed by analysing the induction of the immediate-early gene c-Fos in response to visual stimulation of the deprived eye. The main results of our study were that inhibitory cells did respond to MD, but the neuronal subpopulation expressing PV was refractory to MD maintaining normal levels of c-Fos induction by stimulation of the deprived eye. Similar data were obtained in the cortices contralateral and ipsilateral to the deprived eye. Differently from PV cells, the other inhibitory subpopulations analyzed were affected by MD. The CR expressing neurons displayed reduced visual c-Fos activation after MD, whereas CB cells were the only subpopulation on which MD resulted in a reduction of the marker expression. Most likely the cells that had lost CB expression remained viable although they could be functionally impaired [17]. However, the loss of the CB marker precluded the investigation of c-Fos activation in these cells. Therefore, MD affected neurons belonging to the CB class by altering their neurochemistry and possibly also their functioning, but it is not known whether it also changed their visual responsiveness.

It is generally held that c-Fos is a reliable marker for identifying activated cells and central nervous system circuits that respond to physiological and pharmacological stimuli. Increased neuronal expression of c-Fos results from the complex interplay of several cellular pathways. Stimulation of membrane receptors and subsequent rise in second messengers, including Ca, followed by kinase activation results in the induction of c-Fos expression. Spiking activity is also able to trigger c-Fos expression by increasing Ca levels [13], [14]. Studies on mouse sensory neurons in cell cultures showed that c-Fos expression can be differentially regulated by different patterns of action potentials, and that the temporal dynamics of intracellular signalling cascades are critical in decoding and integrating information contained in the pattern of neural impulse activity [18].

In addition to c-Fos, an immediate-early gene that has also been used to assess the effects of MD is Arc [11]. However, we found that Arc expression was not present in PV cells either in basal condition or after visual stimulation. This result agrees with a previous study [19], showing that Arc does not co-localize with inhibitory neurons in the rat hindbrain. Thus, Arc is not suitable to analyze visually induced gene expression in different populations of inhibitory cells.

### Lack of MD effects on PV cells

Our study indicates that PV cells can be particularly refractory to MD effects. One possible alternative explanation of the persistency of visual c-Fos responses in PV cells of MD rats could be that this cell type can achieve c-Fos expression even in response to the weak activity elicited by visual stimulation of the deprived eye inputs. The nonlinear response of immediate-early genes could then mask the difference between the weak and the strong inputs of the deprived and the nondeprived eye. However, we think that this possibility is unlikely because the direct stimulation of visual cortical slices with different doses of PTX elicited similar c-Fos activation in PV cells and in the other cortical cells. These data indicate that PV cells do not have a particularly low threshold for c-Fos induction. Therefore, the lack of MD effect on c-Fos activation in PV cells is more likely due to a specific response to MD of these cells, or of their afferent inputs, rather than to a different sensitivity of the neuronal activity marker c-Fos in PV cells.

Interestingly, the lack of plasticity of PV cells seems to be rather specific for MD. Although initial studies suggested that PV expression can be regulated by MD, further analyses have indicated that PV expression is not altered by MD [20], [21]. Our data confirm the lack of effect of MD on PV expression and they also demonstrate that visual activation of c-Fos in PV cells is unaffected by MD. By contrast, PV expression and the morphofunctional development of PV cells are responsive to dark rearing or TTX treatment [16], [21]–[23]. This observation suggests that PV cells are able to activate mechanisms of plasticity in response to changes of incoming activity, but that these processes are not activated by MD.

Many indications involve PV cells in regulating the effects of MD on visual cortical circuits. PV cells display a characteristic fast-spiking behaviour depending on the selective expression of the Kv3.1 potassium channel [24]–[26]. Their pattern of innervation of pyramidal cells is suited to control spiking pattern and their reciprocal and extensive connections could regulate the timing of activity in the cortical network [4], [27]. During development, PV positive cells appear in the visual cortex at P11 [28] and continue their maturation during the critical period for OD plasticity [29]. Intriguingly, manipulations that accelerate or delay the time course of the critical period such as dark rearing, BDNF overexpression, removal of polisialic acid, and infusion of Otx2 correspondingly regulate developmental maturation of PV cells [16], [22], [30]–[32]. The alfa1 subunit of the GABA_A_ receptor, that is highly enriched at the postsynaptic site of the basket synapse formed by PV cells on pyramidal neurons, is responsible for the capability of early diazepam treatment to prematurely trigger OD plasticity [33]. Moreover, during late development PV cells become surrounded by components of the extracellular matrix inhibitory for structural plasticity, namely chondroitin sulphate proteoglycans (CSPGs), which condensate in perineuronal nets [34]. Removal of CSPGs from the visual cortex of adult rats promotes visual cortical plasticity [35]. These data suggest that PV cell maturation could be an important determinant of plasticity in the visual cortex, but they do not address how PV cells intervene in the plasticity processes activated by MD. Recent data showed that visual deprivation has opposite effects on PV cells and excitatory star pyramidal cells. Moreover, visual deprivation was shown to induce a cell-specific potentiation in the circuit between fast-spiking inhibitory cells and pyramidal neurons [3], [36]. Thus, visual deprivation can differently affect inhibitory and excitatory circuits, resulting in an alteration of the balance between excitation and inhibition. A modification of the excitatory-inhibitory balance towards inhibition in MD animals could directly contribute to the OD shift induced by MD by depressing cortical responses to deprived eye stimulation [37], [38]. In addition, it has also been suggested that the inhibitory system could filter the activity of the deprived eye inputs favouring plasticity processes leading to their functional disconnection [5], [39]. Indeed, a certain level of GABA inhibition might be necessary to support associative mechanisms of plasticity that rely on the precise timing between pre- and postsynaptic elements [33]. Therefore, a persistent action of the inhibitory system in MD animals could be important also to feed the correct activity pattern into the plasticity mechanisms that underlie the OD shift induced by MD. Our data support these hypotheses by showing that PV cells, at difference from the other cortical cells, preserve visual c-Fos activation in response to stimulation of the deprived eye. Direct measures of visually evoked activity of PV cells in MD animals, albeit technically challenging, are needed to confirm these theories.

In summary, we provide the first *in vivo* evidence that a specific subpopulation of cortical inhibitory interneurons, characterized by the expression of PV, is not affected by long-term MD. These data suggest that PV cells are endowed with specific plasticity mechanisms that could be at the core of their central role in regulating cortical circuit plasticity.

## Materials and Methods

### Animal treatment and surgical procedures

All experiments were performed on Long Evans hooded rats according to the guidelines of the Italian Ministry of Health for care and use of laboratory animals, with housing at 21°C in a 12 h light/12 h dark cycle and food and water *ad libitum*. All the surgical operations were made under avertin anaesthesia (1 ml/hg). Monocular deprivation (MD) was performed at P21 [15]. At an age >P150, MD rats were subjected to the re-opening of the deprived eye, while the previously open eye was closed as in [35]; as a control, animals with previous normal visual experience underwent suture of one eye. Animals were immediately put in a dark light-proof room for 2 days. Afterwards they were re-exposed to light for 2 hours and then intracardially perfused as in Landi et al., 2007 [40]. Coronal sections of 50 µm thickness were cut with a sliding microtome (Leitz, Germany).

### Immunohistochemistry

All the reactions were performed on free-floating sections. After a blocking step in 10% NGS and 0.5 Triton X-100 in PBS (1.5 h at room temperature-RT), double staining for c-Fos and markers of inhibitory cells was done using a PBS-based mix of primary antibodies containing 1% NGS, 0.3% Triton X-100, 1∶3000 rabbit anti c-Fos polyclonal antibody (Calbiochem, USA), and either 1∶1000 anti-parvalbumin (Sigma, Germany) or 1∶1000 anti-GAD67 (Chemicon, USA) or 1∶1000 anti-calbindin (Swant, Switzerland) or 1∶1000 anti-calretinin (Swant, Switzerland) mouse monoclonal antibodies for 36 h at 4°C. Then, we used 1% NGS, 0.1% Triton X-100 with the following secondary antibodies: 1∶400 goat anti-rabbit IgG conjugated to AlexaFluor 488 fluorophore, 1∶400 goat anti-mouse IgG conjugated to AlexaFluor 568 fluorophore for 3 h at RT. For Arc staining we adapted the protocol described in [41]. The primary antibody solution contained 1% NGS, 0.3% Triton X-100, 1∶500 mouse anti-Arc monoclonal antibody (Santa Cruz Biotech., USA) followed by 1% NGS, 0.1% Triton X-100, 1∶400 goat anti-mouse IgG conjugated to AlexaFluor 488 fluorophore, 1∶400 goat anti-rabbit IgG conjugated to AlexaFluor 568 fluorophore (3 h at RT). Slices were mounted on glass slides and covered with VectaShield mounting medium (Vector Labs, USA).

### PTX assay

Rats were decapitated and the brain quickly removed and immersed in an ice-cold cutting solution made as described in [42]. Coronal slices of 350 µm thickness were cut with a vibrating microtome (Leica, Germany) and collected in the same oxigenated cutting solution. Increasing concentrations (0, 0.2, 2, 20 µM) of picrotoxin (PTX) were added to an oxigenated recording solution made as described in [42] and, after incubation for 1 h in the recording solution with different concentrations of PTX, slices were fixed in 4% paraformaldehyde in PB 0.1 M pH 7.4 for 6–8 h, cryoprotected overnight in 30% sucrose in PB 0.1 M pH 7.4, and then cut in 30 µm-thick sections with a Leica cryostat. Free-floating sections were processed for immunohistochemistry as described previously.

### Acquisition and quantification of images

Images from the binocular primary visual cortex (0.501 mm^2^) were acquired at 20× magnification at 1024×1024 pixel resolution using a laser-scanning confocal microscope (Olympus, Japan). Settings for laser intensity, gain, offset and pinhole were optimized initially and held constant through the experiment. For each animal, 5 to 7 slices were acquired and for each section, the three focal planes – spaced 2 µm each one - with the highest signal were acquired. Each channel was acquired in separation to minimize bleedthrough. Images were superimposed with the MetaMorph software (Universal Imaging Corp., USA). Cells positive for each marker or double stained were counted manually and their density was calculated. All analyses were done using a blind procedure.

## Supporting Information

Figure S1 Visually activated Arc does not colocalize with PV. a) Arc positive cells in the cortex contralateral to the visually stimulated eye are significantly increased with respect to the ipsilateral hemisphere (paired t test, P = 0.008); b) average percent of cells showing Arc staining alone (Arc), PV staining alone, or double staining (Arc+PV). Almost no cell showed colocalization between Arc and PV (N = 3); c) low-magnification image of Arc staining in the visually stimulated cortex comprising all cortical layers; d) high-magnification images showing the lack of co-localization between Arc and PV.
(10.43 MB TIF)

Figure S2 MD reduces the number of CB-c-Fos double stained cells after visual stimulation of the deprived eye. Graph showing the decrease in the number of CB and c-Fos double stained cells after long-term MD (t test, P<0.05, N = 10).
(8.14 MB TIF)
