# Supplementary figures and images for: Reduced Responsiveness to Long-Term Monocular Deprivation of Parvalbumin Neurons Assessed by c-Fos Staining in Rat Visual Cortex
Source: PLoS One. 2009 Feb 4;4(2):e4342. doi: 10.1371/journal.pone.0004342 (PMC2632740; doi:10.1371/journal.pone.0004342)

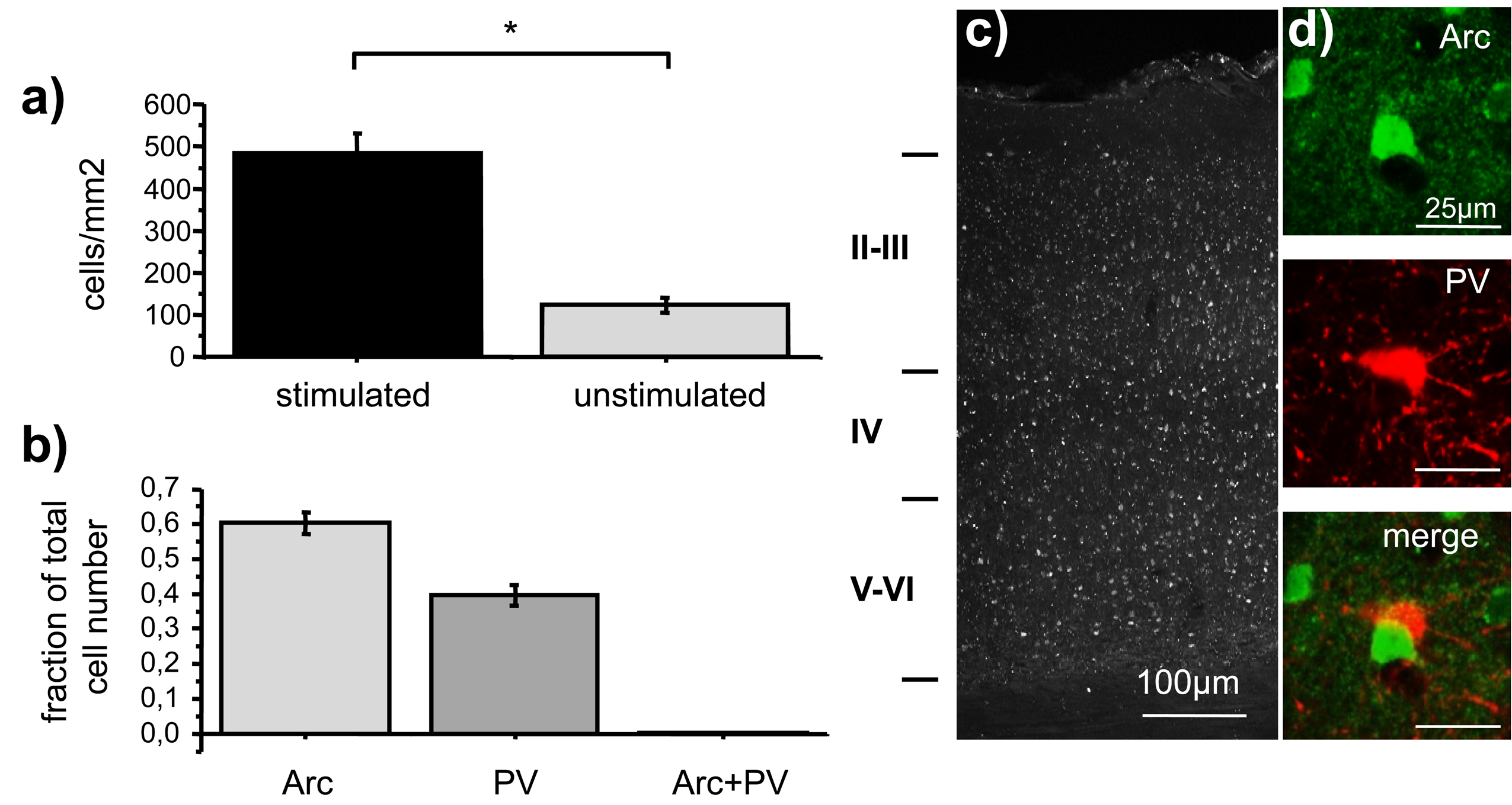

Supplement: Figure S1 — Visually activated Arc does not colocalize with PV. a) Arc positive cells in the cortex contralateral to the visually stimulated eye are significantly increased with respect to the ipsilateral hemisphere (paired t test, P = 0.008); b) average percent of cells showing Arc staining alone (Arc), PV staining alone, or double staining (Arc+PV). Almost no cell showed colocalization between Arc and PV (N = 3); c) low-magnification image of Arc staining in the visually stimulated cortex comprising all cortical layers; d) high-magnification images showing the lack of co-localization between Arc and PV. (10.43 MB TIF) [file pone.0004342.s001.tif]

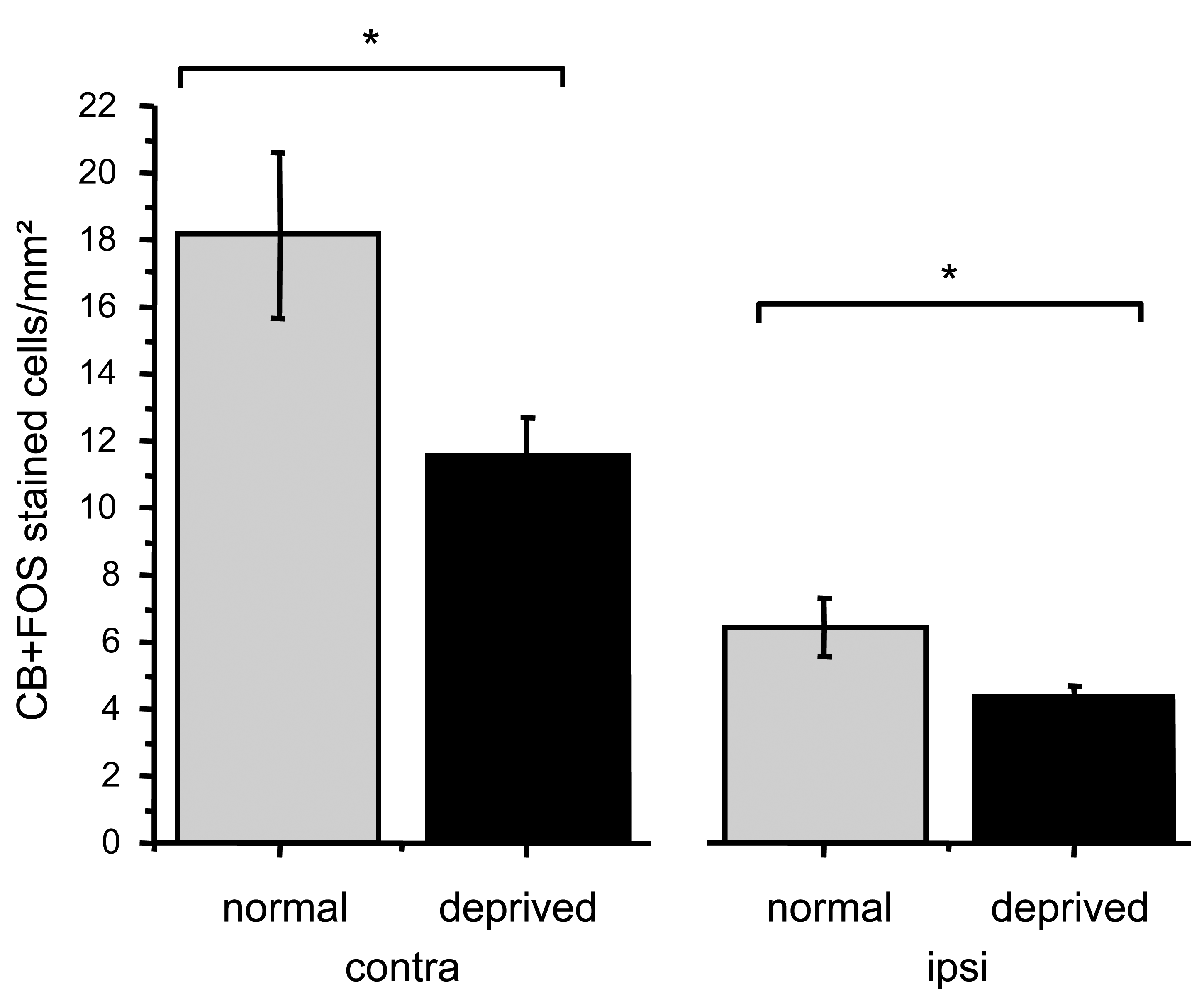

Supplement: Figure S2 — MD reduces the number of CB-c-Fos double stained cells after visual stimulation of the deprived eye. Graph showing the decrease in the number of CB and c-Fos double stained cells after long-term MD (t test, P<0.05, N = 10). (8.14 MB TIF) [file pone.0004342.s002.tif]
